# Atomistic Modelling of Confined Polypropylene Chains between Ferric Oxide Substrates at Melt Temperature

**DOI:** 10.3390/polym8100361

**Published:** 2016-10-14

**Authors:** Ali Gooneie, Joamin Gonzalez-Gutierrez, Clemens Holzer

**Affiliations:** Chair of Polymer Processing, Montanuniversitaet Leoben, Leoben 8700, Austria; joamin.gonzalez-gutierrez@unileobe.ac.at (J.G.-G.); clemens.holzer@unileoben.ac.at (C.H.)

**Keywords:** molecular dynamics, ferric oxide, polypropylene, polymer-metal interactions

## Abstract

The interactions and conformational characteristics of confined molten polypropylene (PP) chains between ferric oxide (Fe_2_O_3_) substrates were investigated by molecular dynamics (MD) simulations. A comparative analysis of the adsorbed amount shows strong adsorption of the chains on the high-energy surface of Fe_2_O_3_. Local structures formed in the polymer film were studied utilizing density profiles, orientation of bonds, and end-to-end distance of chains. At interfacial regions, the backbone carbon-carbon bonds of the chains preferably orient in the direction parallel to the surface while the carbon-carbon bonds with the side groups show a slight tendency to orient normal to the surface. Based on the conformation tensor data, the chains are compressed in the normal direction to the substrates in the interfacial regions while they tend to flatten in parallel planes with respect to the surfaces. The orientation of the bonds as well as the overall flattening of the chains in planes parallel to the solid surfaces are almost identical to that of the unconfined PP chains. Also, the local pressure tensor is anisotropic closer to the solid surfaces of Fe_2_O_3_ indicating the influence of the confinement on the buildup imbalance of normal and tangential pressures.

## 1. Introduction

Mixtures of polymers and metallic particles have numerous applications; for instance, in catalytic and sensing applications [1,2,3,4], and manufacturing techniques [5,6,7]. One interesting manufacturing application is metal injection molding (MIM) or, in more general terms, Shaping, Debinding, and Sintering (SDS) technology of metals. SDS technology consists of (i) preparing a highly-filled mixture of polymers with metallic particles; (ii) shaping this composite by extrusion, injection molding or 3D printing; (iii) removing the polymers; and finally (iv) sintering the metallic particles to obtain a solid metallic part [8]. The behavior of polymers close to a metallic interface can differ drastically from its bulk behavior. Since these systems typically have a high interface density, the behavior of the materials at the nanoscale strongly affects the properties of the system at the macroscale [9]. One instance is during the shaping and debinding steps when the interactions between polymers and metals are crucial to obtain a solid part with the required quality and properties. The polymeric binder should have sufficient affinity to the metallic particles to prevent their agglomeration during the shaping process as well as to prevent excessive distortion of the geometry during the debinding process [10].

Considering the complexity of interfacial systems, one has to resort to computer simulations in many cases in order to study their behavior at different length scales. Computational methods such as atomistic molecular dynamics (MD) [11], Monte Carlo (MC) [12], and dissipative particle dynamics (DPD) [13,14] have attracted a lot of attention in this field. Here, we briefly review some important simulation studies performed on interfacial systems. A number of papers have been devoted to characterize the interfacial properties of small molecules such as water with either their vapor or solid surfaces. The surface tension of equilibrated liquid-vapor water was simulated using MD at temperatures from 316 to 573 K [15]. This system was later revisited by Shi et al. [16] utilizing the particle-particle particle-mesh method. More recently, Fernandes et al. [17] and Negreiros et al. [18] utilized MD simulations to study the properties of water on iron and iron oxide surfaces, respectively. Other materials investigated via MD simulations include iron surfaces in the presence of ionic liquids [19] and solid-liquid metals [20].

Due to their practical importance, more complex materials such as polymers have also been simulated to study their interaction with different substrates. In 1991, Mansfield et al. [21] performed MC and MD simulations to study the adhesion of atactic polypropylene (PP) to graphite. Matsuda et al. [22] performed MD simulations of *n*-alkane melts at temperatures of 300 K and 400 K confined by either neutral or attractive generic crystalline surfaces. The work of Dauolas et al. [12] presented an atomistic modelling approach to simulate the interface between a thin film of polyethylene (PE) melt supported by a semi-infinite graphite substrate. Afterwards, Harmandaris et al. [23] extended their work to study the local dynamics and chain mobility of a thin film of molten PE at 450 K adsorbed on the crystalline phase of graphite. Larsen et al. [24] performed MD simulations combined with DPD to predict the homogeneity of iron-nickel metal powder in a mixture of PP, paraffin wax, and stearic acid. In order to study the cross-linked structure of epoxy near an alumina surface, Kacar et al. [25] developed interfacial MD models which were then coarse-grained into DPD models. Anastassiou and Mavrantzas [26] performed MD simulations for poly(*n*-butyl acrylate) and poly(*n*-butyl acrylate-*co*-acrylic acid) on α-quartz, α-ferric oxide, and α-ferrite. Liu et al. [27] studied via MD the interfacial region of surface modified cadmium sulfide in contact with mercaptopropyltrimethoxysilane. MD simulations were carried out by Ta et al. [28] to observe the structural properties and adsorption energies of alkanes on highly relaxed iron and its oxide surfaces. Abraham et al. [29] presented a MD scheme to study the time evolution of gold clusters sputter-deposited on a planar surface of polystyrene. A number of studies as well as remaining challenges related to atomistic, coarse-grained, and multiscale simulations of interfacial systems of polymers and biomolecules with substrates such as silicates, glasses and oxides have been summarized in the literature reviews of Johnston and Harmandaris [9] and Heinz [30].

Two important materials in MIM are PP and ferritic alloys such as steel. PP with high molecular weight is generally used as the backbone in the binder system. Ferritic alloy particles tend to form a ferric oxide (Fe_2_O_3_) layer on their outermost surface as a result of the air oxidation occurring at most temperatures even if chromium is present, such as in stainless steel [31]. The composite materials used in MIM are highly-filled systems with a minimum of 50 vol % metallic particles. For these reasons in this paper we investigate the interfacial properties of molten PP chains confined between two Fe_2_O_3_ substrates incorporating atomistic MD simulations. The work performed here is unique since, to our knowledge, there are no MD simulations directly dealing with molten PP at 458 K confined by high-energy Fe_2_O_3_ surfaces. Moreover, we have adopted an optimized atomistic force field, the INTERFACE force field, particularly parametrized for the organic-inorganic interfaces by Heinz et al. [32]. This force field has proven effective to study a number of interfacial systems including metals [33]. Consequently, the interactions between low-energy PP and high-energy Fe_2_O_3_ is modelled based on a solid theoretical reference making it suitable for structural analyses.

## 2. Models and Methods

### 2.1. Molecular Model

The system was composed of atactic PP chains confined between two Fe_2_O_3_ substrates. Each PP chain consisted of 50 monomers designed with a head-to-tail orientation. Considering that the number of monomers in a realistic PP chain is significantly more than 50, one should expect differences between the simulation results and the real systems. Throughout the paper, we attempt to address such differences. A total number of 10 chains was considered in the simulations. This number of chains was found to be sufficient to produce a fully-developed symmetrical local density profile in the film thickness with a mass density equal to the unconfined polymer systems in the middle of the film. Fe_2_O_3_ was chosen in this study due to its industrial and practical significance in the SDS process, as explained before. Each Fe_2_O_3_ substrate was composed of 750 atoms ordered in a face-centered cubic (fcc) crystalline structure [34]. The crystal was re-produced in an orthogonal cell with dimensions *L*_x_ = *L*_y_ ≈ 27.4 Å and *L*_z_ ≈ 9.61 Å. An all-atom description was used for the materials which made it possible to acquire quantitative predictions of the relevant properties of the confined system.

The molecular model was based on the INTERFACE force field developed by Heinz et al. [32,33]. This thoroughly parametrized interfacial force field is specifically optimized for organic-inorganic interactions using experimental material properties such as density and surface energies as target properties to fit the Lennard-Jones (LJ) atomic radii and well depth. INTERFACE is a fixed-charge force field [35] which is built upon the common harmonic force fields in materials science such as PCFF [36], CHARMM [37], and COMPASS [38]. The successful applications of this force field to a range of materials including clay minerals [39], fcc metals [33], polymers [27,32], and biomolecules [30] motivated its use in the current system.

The main contributions to the total potential energy of the system in INTERFACE arise from nonbonded interactions, bond stretching, bond-angle bending, dihedral torsions, and improper interactions. INTERFACE utilizes Class II definitions to describe these contributions. The force field also contains energy contributions from the extra terms in Class II including bond-bond interactions, bond-angle interactions, angle-angle interactions, angle-angle torsions, end- and middle-bond torsions, and angle torsions [32]. The main achievement of the INTERFACE was the modification of the nonbonded parameters for the 9-6 LJ potential [33] $E_{\mathrm{LJ}}(r)$ of the form

(1) $$E_{\mathrm{LJ}}(r)=\varepsilon \left[2{\left(\frac{r_{0}}{r}\right)}^{9}-3{\left(\frac{r_{0}}{r}\right)}^{6}\right]\;\mathrm{for}\;r\leq r_{c},$$

where ε is the equilibrium nonbonded energy, and $r_{0}$ is the equilibrium nonbonded distance between two atoms of the same type. In this equation, $r_{c}$ is the cutoff distance which was set to 12 Å in all simulations. Heinz et al. [33] showed that the modified potentials are capable of providing more accurate predictions for the super cell parameters of a number of metals in comparison with conventional potentials. The same improvement was also observed when the surface tensions of metal-water systems were calculated and compared with the experimental values [33].

A Coulomb potential was also included in the model as

(2) $$E_{C}(r)=\frac{q_{i}q_{j}}{4\pi \varepsilon_{0}r},$$

in order to describe electrostatic interactions associated with partial atomic charges q. The partial charges for PP atoms were calculated utilizing the Gasteiger method [40] of partial equalization of orbital electronegativity. The atomic charges of atoms in Fe_2_O_3_ crystals were set to 1.2 and −0.8 (in units of e^−^) for Fe and O atoms, respectively. These values were based on the work of Batista et al. [41] who developed a self-consistent charge-embedding methodology for ab-initio quantum chemical modelling of Fe_2_O_3_ crystals with various sizes. The electrostatic interactions were included in the simulations using the particle–particle particle–mesh (PPPM) method with a real space cutoff of 12 Å.

The INTERFACE force field is well described in the paper of Heinz et al. [32] and its parameters are available for public use. Consequently, we do not delve further into its details and refer the interested reader to the cited publication and its supplementary information.

### 2.2. Simulations Methodology

**Figure 1:**
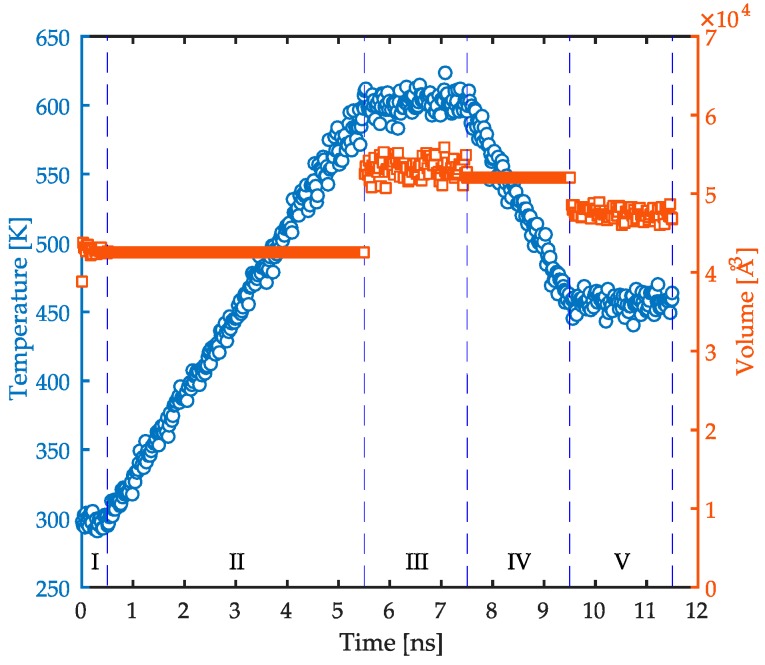
The temperature (**circles**) and volume (**squares**) of the system during the relaxation process as a function of time. Each stage of the process is numbered and separated with vertical dashed lines.

Detailed atomistic MD simulations were performed with model PP/Fe_2_O_3_ samples in a *xyz* Cartesian coordinate system using the molecular model described in the previous section. All of the simulations were carried out with the LAMMPS (Sandia National Laboratories, Livermore, CA, USA) [42] (large-scale atomic/molecular massively parallel simulator) code package. In order to build the confined films, an orthogonal amorphous cell of PP chains was initially developed based on the method of Theodorou and Sutter [43,44] with dimensions of *L*_x_ = *L*_y_ ≈ 27.3 Å and *L*_z_ ≈ 66.5 Å. This system consists only of polymer macromolecules and is used to run relaxation simulations before simulating the confined system. Therefore, it can be considered as a bulk simulation. The simulation box was subject to periodic boundary conditions in all three space directions. We used a specific simulation protocol to achieve the equilibrated polymer configurations which includes heating and cooling stages, see Figure 1. The relaxation process started with an initial energy minimization of the cell. Afterwards, a dynamic simulation in the NPT ensemble was performed at 298 K and 1 atm for 0.5 ns (stage I in Figure 1). Next, the system was heated up in a NVT ensemble from 298 to 600 K in 5 ns (stage II in Figure 1). Then, the system was allowed to relax at 600 K and 1 atm for 2 ns in a NPT ensemble (stage III in Figure 1). Afterwards, the temperature was reduced to 458 K with a cooling ramp in 2 ns in a NVT ensemble (stage IV in Figure 1). Next, the system was relaxed for another 2 ns in a NPT ensemble at 458 K and 1 atm (stage V in Figure 1). Finally, an energy minimization was performed with the Polak-Ribiere version of the CG algorithm [45]. The purpose of relaxation at high temperatures was to accelerate the molecular relaxation processes, and avoid trapping in high-energy minima [45]. It should be noted that the density of the system reached a steady value of 0.737 ± 0.009 g·cm^−3^ in the final stage of relaxation. This value is in good accordance with the typical experimental values for PP at 458 K and 1 atm.

**Figure 2:**
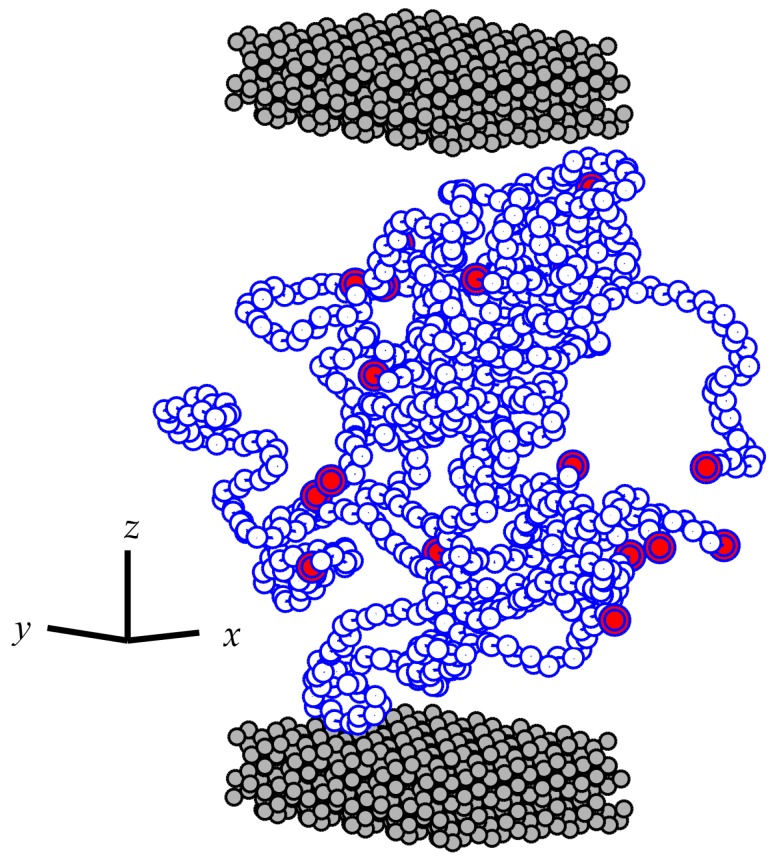
Schematic representation of the simulated confined systems. In the figure, only the backbone carbon atoms of the chains are shown to preserve clarity. The two ending carbon atoms of each PP chain are plotted in slightly larger circles filled with **red** color. The substrates are shown in **black** circles filled with gray color at the **bottom** and **top** of the simulation box.

The equilibrated PP configurations from the relaxation process were then sandwiched between the Fe_2_O_3_ substrates along the *z* direction. This procedure inevitably changes the density of the confined polymer film from that of the bulk simulations. To compensate for this problem, the confined system was allowed to thoroughly relax prior to data collection. The relaxation procedure is described in detail later in this section. After this relaxation procedure, the film had the dimensions of *L*_x_ = *L*_y_ ≈ 27.4 Å and *L*_z_ ≈ 64.7 Å. It was noted that this relaxation step was effective since the density profile of the confined polymers showed comparable values in the middle region of the film to that of the unconfined bulk simulation. This behavior is also a manifestation of the fact that the total number of polymer chains with 50 monomers used here can successfully reproduce a large enough thickness so that the middle region of the film is structurally the same as the unconfined polymers in the bulk. A snapshot of the simulated confined system is shown in Figure 2. It should be noted that the average value of the radius of gyration of polymer chains was calculated to be 14.16 ± 0.08 Å during the final stage of the relaxation cycle. A comparison of this value with the thickness of the film provides insight into how confined the chains are. The thickness of the film is almost 4.6 times the average radius of gyration of the chains. This difference was found adequate to have a fully-developed density profile across the thickness of the film. Such a well-developed density profile has been shown by other authors to be a valuable tool for coarse-graining atomistic models [25,46]. The coarse-graining allows incorporating simpler forms of potential energy and consequently perform simulations on longer time scales [47]. Therefore, the degree of confinement in this study provides us with the possibility of a multiscale simulation of highly-filled composites. This issue shall be addressed in a future publication.

In order to perform the interfacial simulations, an energy minimization was initially performed on the polymer chains of the confined system. Afterwards, thermal equilibration was achieved by performing dynamic simulations in NPT ensemble at 458 K and 1 atm for 10 ns. This step allowed for the adjustment of the density of the polymer bulk after the sandwiching procedure by allowing the system to reach a steady volume under the desired thermodynamic conditions. Next, the system was simulated in a NVT ensemble at 458 K for another 10 ns to ensure stable interfacial structures are developed for the calculations. Finally, the data collection step included a dynamic simulation run in a NVT ensemble at 458 K for 20 ns during which the desired structural and thermodynamic properties were averaged over time as well as all PP configurations. The use of the NVT ensemble in the data collection step allowed us to have a constant degree of confinement by keeping the volume constant. Moreover, the fundamental concepts of such interfacial systems are mainly developed in the NVT ensemble [44,48]. The NVT ensemble has also been widely used in a variety of interfacial simulations to investigate the thermodynamic properties [16,18,22,28,49]. During all simulation steps, the simulation box was subject to periodic boundary conditions in all three space directions.

In all simulations performed in the NPT statistical ensemble, a Nosé-Hoover thermostat-barostat [50,51] was employed to control temperature and pressure with corresponding relaxation times of 100 and 1000 fs, respectively. For the simulations in NVT ensemble, a Nosé thermostat was used with a relaxation time of 100 fs. The equations of motion were integrated using the velocity–Verlet algorithm [52,53] with a time step of 1 fs.

## 3. Results and Discussion

### 3.1. Local Density Profile

**Figure 3:**
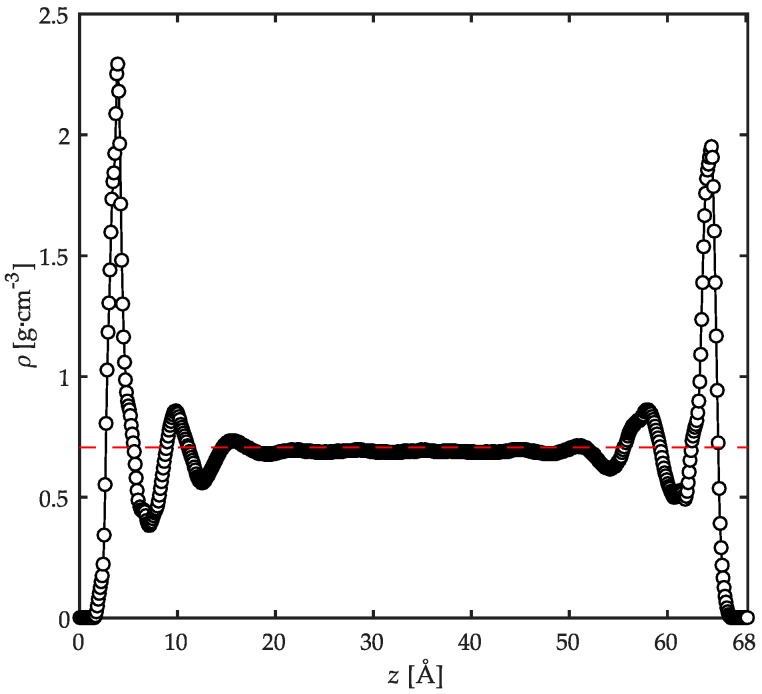
Local mass density profile, ρ, across the film thickness. The horizontal dashed line represents the mass density of the polymer from the bulk simulations. The results are calculated for the confined PP/Fe_2_O_3_ system at 458 K and 1 atm.

The local density profile of PP chains in the film thickness was analyzed by dividing the simulation box in the *z* direction into bins separated by planes parallel to the substrates’ surfaces each of width equal to 0.1 Å. The average of the local density profile was then computed within each bin over all chain configurations. Figure 3 displays the mass density profile, ρ, across the film thickness. Due to the symmetry of the density profiles with respect to the film midplane, one can conclude that the number of polymer configurations embedded in the film thickness was sufficient to provide adequate predictions of the relevant properties studied here [26]. Near the substrates, the density profile shows a profound peak at a distance equal to ~4 Å away from the solid surface. This distance is approximately equal to the sum of the van der Waals radii of polymer and substrate atoms. Moving away into the bulk of the film, a minimum appears roughly at 7 Å away from the solid surface, representing the thickness of the adsorbed layer of PP macromolecules on the substrate. A second adsorption peak, much less pronounced than the first peak, appears roughly at 10 Å with a corresponding minimum at ~12.5 Å, representing the second polymer layer adjacent to the solid surfaces. At distances beyond 20 Å away from the solid surface, the density attains values equal to the unconstrained polymer bulk density calculated in the relaxation simulations. This suggests that in this region the structure of PP chains are almost identical to those in unconfined simulations. For polyethylene chains with various numbers of backbone carbon atoms (from 40 up to 250) adsorbed on a graphite substrate at 450 K, the thickness of the interfacial region was also calculated to be 20 Å which is in agreement with the present work [12]. In a separate report, Mansfield and Theodorou [54] calculated an interfacial thickness of 10 Å for PP with 76 monomers in each chain on graphite at 233 K. The authors point to the fact that this thickness is significantly smaller than the overall chain dimensions. Based on these works, one can draw the conclusion that the thermal motions play a critical role in the determination of the interfacial thickness as opposed to the chain dimensions [48]. However, the adsorbed chains still contribute to the overall density profile depending on their length, as will be discussed later. Moreover, the chain dimensions influence the bulk density value in the middle of the film to some extent [12].

The formation of the adsorbed PP layer on the substrates in this confined system is simultaneously affected by energy minimization and space-filling considerations. The very attractive interactions (electrostatic and van der Waals) developed between PP atoms and atoms of the two oxides result in well-ordered atomic structures next to the solid substrates. These structures are manifested by the sharp peaks in the local density profile. For less favorable interactions, these peaks would be reduced significantly. Such observations have been shown before for the interfacial systems of acrylic chains on α-quartz, α-Fe_2_O_3_, and α-ferrite where the strong adsorption of acrylic chains on α-quartz and α-Fe_2_O_3_ was evidenced by the higher intensities of the local mass density peaks compared with the less pronounced peaks for α-ferrite [26]. These peaks are, on the other hand, influenced by the available space on the surfaces. The maximum packing of atoms on the surfaces is strongly controlled by the steric hindrances from surrounding atoms. The peaks in the local density profile have consequently a maximum value above which no more adsorption of atoms is possible due to the shortage of free space. As will be discussed later, this notion gives rise to some irregularities in the local conformation of chains and bonds as well.

Finally, it is important to note the well-equilibrated polymer chains in this confined configuration as evidenced by the smooth density profile in the middle of the film. This behavior is a result of the high temperatures used in the relaxation procedure and the simulations as opposed to the noisy density profiles in low-temperature simulations reported in a previous work [26].

**Figure 4:**
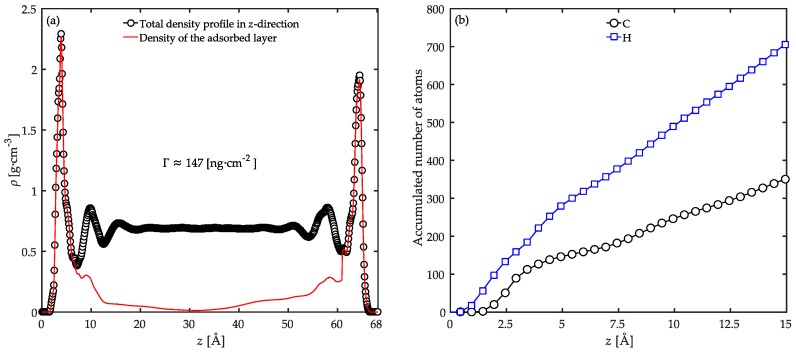
(**a**) Local contributions to the total mass density profile (**open circles**) from segments of the first polymer layer adjacent to the substrates (**red continuous line**); (**b**) The accumulated number of hydrogen and carbon atoms across the film thickness calculated from the number density profiles of the corresponding atoms. The results are calculated for the confined PP/Fe_2_O_3_ system at 458 K and 1 atm.

An interesting analysis of such a confined system is to characterize the extension of interfacial chains into the bulk of the film. In order to investigate such effects, we calculated the local contribution of polymer segments from the adsorbed polymer layer on the substrates to the total mass density profile. The results are demonstrated in Figure 4a. One can observe that the polymer chains in the adsorbed layer (the interfacial chains) contribute to the density, and consequently influence the structure of the film to a larger extent, approximately up to 25 Å into the bulk of the film. This means that any polymer chain with at least one atom located at the interfacial region with the substrate (within 7 Å away from the solid surface) is limited within a layer of 25 Å width inside the film next to the surface. This adsorbed segment density profile at distances larger than the limits of the interfacial region is due to the presence of the long dangling tails. It is obvious from this result that the length of the chains determines the depth by which an adsorbed layer affects the local density profile. Considering that the chains used in our simulations are significantly smaller than in reality, one should expect a much larger influencing range for the adsorbed chains in practice.

The formation of adsorbed monolayers on substrates has been reported before for *n*-alkane molecules [22,28,55]. However, a difference between their work and the work reported here is that they deal with monolayers of short chains next to a substrate, while we deal with a bulk phase of long chains. In our case, the balance between adsorption energy, cohesive energy, and conformational entropy is struck at a much less uniform distribution of conformations. This point was also stated in the work of Mansfield and Theodorou [54]. Consequently, the definition of adsorption in terms of monolayers is not possible since the adsorbed atoms do not form a uniform layer on the surface. This issue was also illustrated in the works of Anastassiou et al. [26]. and Daoulas et al. [12].

Following Scheutjens and Fleer [56,57], the adsorbed amount, Γ, was approximated by integrating the local density profile of the first adsorbed layer to be equal to ~147 ng·cm^−2^. In a previous study, Daoulas et al. [12] performed MD and MC simulations of PE chains with various numbers of carbon atoms in their backbone adsorbed on a graphite substrate. Their estimation for the adsorbed amount of the PE chains with 100 backbone carbon atoms (the same as PP chains used in this study) was approximately in the order of ~110 ng·cm^−2^. This difference in the adsorbed amounts could be ascribed to different surface energies of the graphite and Fe_2_O_3_ substrates. One should note that the polymer model employed here is all-atomistic while in the work of Daoulas et al. [12] a united-atom approach was utilized to represent methylene and methyl groups. Therefore, a more precise balance between energetic and entropic interactions of polymer conformations should be achieved in our models. On one hand, the steric hindrance of the pendant methyl groups on PP goes against the formation of compact adsorbed structures at the interface. On the other hand, the higher adsorption energy of the hydrophilic Fe_2_O_3_ surface promotes the adsorption process. This conflict controls the number of adsorbed atoms on the surface of Fe_2_O_3_. The accumulated number of hydrogen and carbon atoms from the surface of the lower substrate was calculated from the number density profiles of the corresponding atoms. Figure 4b shows the evaluated accumulated number profiles. The number of adsorbed hydrogen and carbon atoms in the interfacial region of the lower substrate is approximately 356 and 172, respectively. These values sum up to an adsorbed amount of ~106 ng·cm^−2^, which is more than 70% of the total amount. Consequently, the balance between the steric hindrance of side groups of PP and the adsorption energy of Fe_2_O_3_ surface is strongly in favor of interfacial adsorption. Therefore, the length of the chains could become the determining factor in the adsorbed amount only when the surface energy of the substrate is not dominant. Still, the significance of the chain size should not be ignored in the adsorbed amount of polymer, the structure formation of the adsorbed layer as well as the bulk of the film.

### 3.2. Conformational Characteristics of the Confined Film

**Figure 5:**
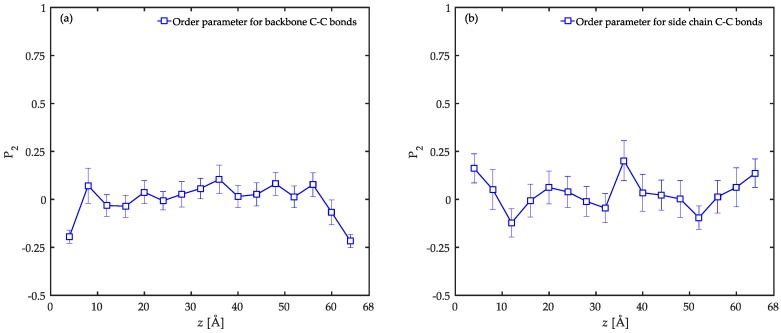
Local order parameter ($P_{2}$) for C–C bonds in (**a**) backbone and (**b**) with side chains as a function of distance from the surface of the substrate. The results are calculated for the confined PP/Fe_2_O_3_ system at 458 K and 1 atm.

The presence of solid substrates induces local re-arrangements in the polymer film particularly at the interface. In order to provide insight into such characteristics of the film, the average second-rank bond order parameter $P_{2}$ was calculated according to

(3) $$P_{2}=\frac{3}{2}\langle \cos\theta \rangle -\frac{1}{2},$$

where θ is the angle formed between the bonds and the *z* coordinates axis. This parameter varies between three limits of −0.5, 0.0, and 1.0 which correspond to perfectly parallel, random, and perfectly normal orientation states relative to the solid surface, respectively. The order parameter for C–C bonds either in the backbone or with the side group of PP chains is shown in Figure 5. In these calculations, the film thickness was divided into bins each with 4 Å width and the bonds were assigned to bins according to the distance of their midpoints from the bins. For C–C bonds of the backbone, a parallel alignment is observed in the interfacial regions. In these regions, a slight tendency towards normal orientation with respect to the solid surface is observed for the C–C bonds with the side chains according to the data. This behavior is strongly influenced by the instantaneous local perturbations due to the small length of this type of C–C bonds [26]. For both C–C bond types, the local orientation parameter oscillates around zero in the bulk of the film further away from the substrates, and thus manifests random orientation states dominant in those regions. Similar trends have been reported before for atactic PP with an interface with a graphite substrate [54], PE confined between graphite substrates [12], and acrylic adhesives between either α-quartz or α-Fe_2_O_3_ substrates [26].

The local orientation of C–C bonds is significantly influenced by the available space on the solid substrates. In the interfacial system of poly(*n*-butyl acrylate)/α-Fe_2_O_3_, the order parameter for the backbone C–C bonds shows a more pronounced parallel orientation in the interfacial region ($P_{2}$ values closer to −0.5) than our simulations [26]. In the cited work, the dimensions of the solid surface are reported to be *L*_x_ = 68.9 Å and *L*_y_ = 70.5 Å while in our case they are *L*_x_ = *L*_y_ ≈ 27.3 Å. Therefore, there is almost six times more available surface for the polymer chains in the poly(*n*-butyl acrylate)/α-Fe_2_O_3_ system. In this system, the calculated interfacial energy is ~1654 mJ·m^−2^ while in our system it is calculated to be ~1180 mJ·m^−2^ (the calculation is given in detail in the next section). Thus, one might ascribe the differences in the values of the order parameters to energy considerations. In order to show the importance of the available space rather than the energy considerations, we took the system of PP chains adsorbed on a graphite substrate as a valuable example [54]. In this system, the interfacial energy was simulated to be ~116 mJ·m^−2^ with the corresponding dimensions of the substrate to be *L*_x_ = 24.6 Å and *L*_y_ ≈ 25.6 Å [54]. In this work, the order parameters in the interfacial region were calculated to be very close to the values of our system. As a result of these arguments, the available space rather than the energy considerations is shown to be the determining factor in the local conformation of the adsorbed chains in the interfacial region.

**Figure 6:**
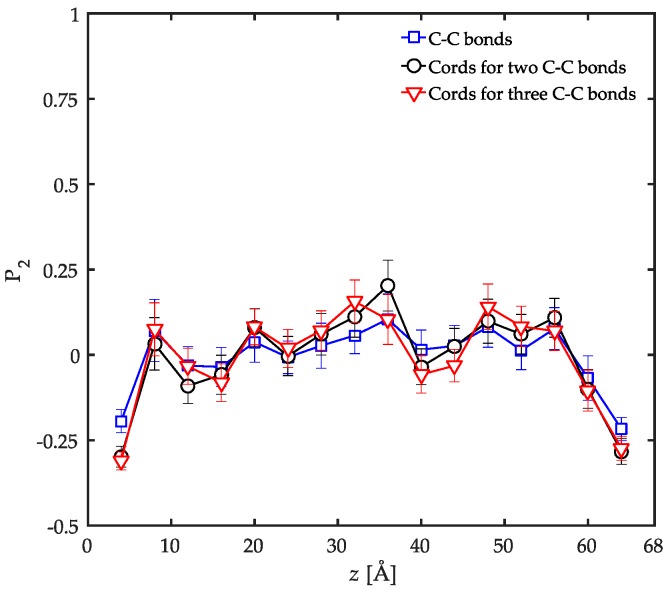
Local order parameter ($P_{2}$) as a function of distance from the surface of the substrate for C–C bonds of the backbone (squares), cords connecting two C–C bonds (circles), and cords connecting three C–C bonds (triangles). The results are calculated for the confined PP/Fe_2_O_3_ system at 458 K and 1 atm.

The orientation state of the bonds could simply vary at different length scales due to the fact that the orientation process on the small length scale of a C–C bond is instantaneously perturbed by thermal fluctuations. Therefore, the order parameter was evaluated on slightly larger length scales than a single C–C bond so that the influence of such perturbations was studied. This was achieved by replacing every two or three C–C bonds along the backbone with a single hypothetical cord and evaluating $P_{2}$ for these cords instead of individual bonds as a function of distance from the surface of the substrate. The results are compared with single C–C bonds in Figure 6. While all orientation profiles display random chain orientation in the bulk of the film, the preferred parallel ordering in the interfacial regions becomes more pronounced only slightly on increasing the length of the cords. Therefore, the orientation state of the bonds is not significantly influenced by increasing the length scale in the limited range of a few C–C bonds. Thus, the local perturbations are expected to be immensely suppressed due to the strong tendency of the surfaces to adsorb atoms. This issue was verified before by the analysis of the adsorbed amount. The data is also an indication that the local ordering along the chains at the interfaces is persistent at scales up to three C–C bonds. It should be declared that these results cannot help disclose local ordering on larger scales.

**Figure 7:**
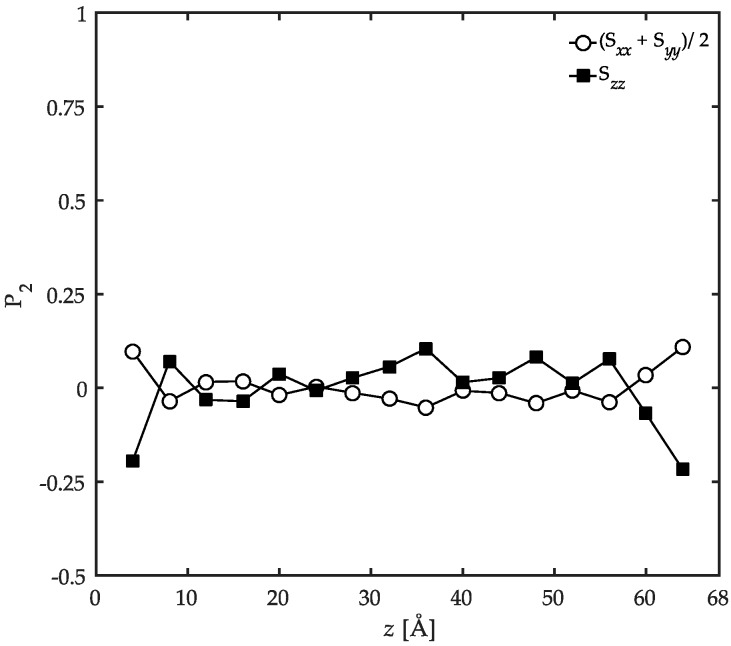
Values of the diagonal components of the Saupe matrix, $S_{\mathrm{ab}}$, as a function of *z* coordinate. The results are calculated for the confined PP/Fe_2_O_3_ system at 458 K and 1 atm.

The anisotropy of the oriented structure can be analyzed based on the symmetric Saupe matrix [12], S, as

(4) $$S_{\mathrm{ab}}=\frac{3}{2}\langle l_{a}l_{b}\rangle -\frac{1}{2}\delta_{\mathrm{ab}},$$

where $l_{i}$*s* with i=a, b are the direction cosines relating backbone C–C bonds to the box Cartesian coordinate frame. If the polymer film is characterized by the uniaxial anisotropy in bond ordering along the *z* direction in our confined systems, all off-diagonal components of the Saupe matrix should go to zero while the main diagonal components should satisfy the condition $S_{\mathrm{xx}}{=S}_{\mathrm{yy}}=-\frac{1}{2}S_{\mathrm{zz}}$. This condition was checked in the simulations performed here and the results are reported in Figure 7. The uniaxial ordering character in the *z* direction is satisfied within the statistical accuracy of the simulation data. This observation denotes that the orientation of the backbone C–C bonds in the *z* direction (normal to the solid surfaces) is systematic and of high significance while their orientation state in the *xy*-plane is not. Similar to this work, a uniaxially-ordered configuration was also predicted in the interfacial polyethylene melts at 450 K on a graphite substrate using Monte Carlo simulations [12].

The overall alignment of a chain, either parallel or perpendicular to the solid surface, was estimated benefitting from the conformation tensor, $C^{0}$. This tensor can be regarded as a global descriptor of the overall polymer melt configuration. It is defined as the second moment tensor of the end-to-end distance vector of a polymer chain R reduced by one third of its unperturbed mean-square end-to-end distance $\langle R_{0}^{2}\rangle$ averaged over all chain configurations, as

(5) $$C^{0}=3\langle \frac{RR}{\langle R_{0}^{2}\rangle }\rangle .$$

**Figure 8:**
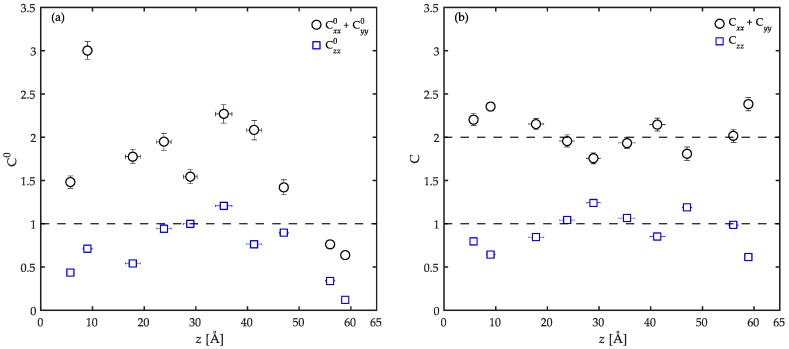
The overall alignment of PP chains along the thickness of the film either parallel ($C_{\mathrm{xx}}^{0}+C_{\mathrm{yy}}^{0}$) or normal ($C_{\mathrm{zz}}^{0}$) to the substrates expressed utilizing the concept of the conformation tensor. The results in (**a**) are calculated based on Equation (5); in (**b**), Equation (5) was reduced by the end-to-end distance of each confined chain instead of the averaged end-to-end distance of the unperturbed chains. Therefore, the ‘0’ superscript was removed from the notations of the conformation tensor and its components to emphasize this distinction. The data are calculated for each chain individually and are plotted as a function of the *z* coordinate of the center of mass of that respective chain. Note that the horizontal error bars are multiplied by a factor of 10 to increase their visibility. The results are calculated for the confined PP/Fe_2_O_3_ system at 458 K and 1 atm.

For an unperturbed chain, $C^{0}$ reduces to the unit tensor. However, any distortion in the chain configuration as a result of the confinement should impose deviations in $C^{0}$ from its isotropic value. In this case, the three main diagonal components of the conformation tensor, i.e., $C_{\mathrm{xx}}^{0}$, $C_{\mathrm{yy}}^{0}$, and $C_{\mathrm{zz}}^{0}$, can provide a measure of orientation and extension of the chains along the three axes of the coordinate system. In order to find the tendency of a chain to lie in the *xy*-plane parallel to the substrate surface, one can plot the sum of the contributions in the *x* and *y* directions to the conformation tensor, i.e., $C_{\mathrm{xx}}^{0}{\;+\;C}_{\mathrm{yy}}^{0}$. On the other hand, $C_{\mathrm{zz}}^{0}$ can represent the perpendicular orientations with respect to the solid surfaces. The results of such analyses are reported in Figure 8a. The components of the conformation tensor are calculated for each chain individually and plotted as a function of the *z* coordinate of the center of mass of that respective chain. It is clear that the chain conformations across the film are significantly different from their unperturbed shape in the bulk with no confinement. It is evident from the data that the chains located closer to the substrates are extensively compressed along the *z* direction. These chains show a higher tendency to lie in *xy*-planes parallel to the surface ($C_{\mathrm{xx}}^{0}{\;+\;C}_{\mathrm{yy}}^{0}{>C}_{\mathrm{zz}}^{0}$). However, it does not always lead to extended chain conformations in the *xy*-plane ($C_{\mathrm{xx}}^{0}{\;+\;C}_{\mathrm{yy}}^{0}<1$ close to the upper substrate) which can be ascribed to strong adsorption of the chains on the surfaces prohibiting large-scale structural re-arrangements. The extremely small horizontal error bars in these regions of the film can be an indication of such strong adsorptions on the solids. This adsorption effectively prohibits any exchange of chains between the adsorbed layer and the bulk of the film. Otherwise, the horizontal error bars were much more significant than in the present case. Note that the horizontal error bars are multiplied by a factor of 10 to increase their visibility in the figure. As one moves away from the solid surfaces into the bulk of the film, the chain compression in the *z* direction disappears with $C_{\mathrm{zz}}^{0}$ reaching to unity. This suggests that in the bulk of the film the configurations of the chains mimic that of the unconfined chains in the *z* direction. In parallel *xy*-planes however, the chains are still extended and deviate from the unperturbed conditions ($C_{\mathrm{xx}}^{0}{\;+\;C}_{\mathrm{yy}}^{0}>1$).

Daoulas et al. [12] simulated the components of the conformation tensor for polyethylene melts at 450 K on a graphite substrate. At distances of 10, 20, and 30 Å away from the graphite surface the corresponding components of the conformation tensor are respectively $C_{\mathrm{zz}}^{0}\;\sim \;0.2$ and $C_{\mathrm{xx}}^{0}{\;+\;C}_{\mathrm{yy}}^{0}\;\sim \;3$, $C_{\mathrm{zz}}^{0}\;\sim \;0.5$ and $C_{\mathrm{xx}}^{0}{\;+\;C}_{\mathrm{yy}}^{0}\;\sim \;1.9$, and finally, $C_{\mathrm{zz}}^{0}\;\sim \;0.9$ and $C_{\mathrm{xx}}^{0}{\;+\;C}_{\mathrm{yy}}^{0}\;\sim \;2.2$. These values are in good agreement with our results at distances further into the bulk. At the interfacial regions, however, the overall chain sizes in our simulations are smaller than Daoulas et al. [12]. Taking into account the previous arguments, this observation could be ascribed to space-filling considerations. Daoulas et al. [12] performed their simulations on a substrate surface with *L*_x_ = 49.2 Å and *L*_y_ ≈ 46.9 Å which provides a surface area of approximately three times more than our simulations. Furthermore, as it was argued before, their results are reported for a coarse-grained polyethylene melt with 78 backbone carbon atoms. This also implies a smaller chain with less steric hindrance which was brought to contact on a larger graphite surface in comparison with the present model. Consequently, the importance of the available space for the development of conformations persists on a larger scale than bonds and influences the flattening of the chains as well.

In order to have a clearer picture of how flat a chain is, Equation (5) was reduced by the end-to-end distance of each confined chain instead of the averaged end-to-end distance of the unperturbed chains. This should allow us to rationalize the contributions of each direction of the coordinate system to the overall chain end-to-end distance. In this way of description, the more a chain is flattened in the *xy*-plane the less its corresponding *zz* component is in the conformation tensor. In order to emphasize the distinction in the two definitions of the conformation tensors used here, the “0” superscript was removed from the notations of the conformation tensor and its components. The results are presented in Figure 8b. It is obvious from the data that the chains prefer to flatten in the *xy*-plane as they all have larger $C_{\mathrm{xx}}{\;+\;C}_{\mathrm{yy}}$ values compared with $C_{\mathrm{zz}}$. This flattening results in the overall parallel orientation of the chains with respect to the substrate surfaces. Moreover, it is important to note that the chains which are located at the interfacial regions show a more pronounced parallel orientation than those in the bulk due to the relatively larger difference between $C_{\mathrm{xx}}{\;+\;C}_{\mathrm{yy}}$ and $C_{\mathrm{zz}}$ values in these regions compared to the bulk of the film.

Based on the information provided by the local order parameter ($P_{2}$) and the conformation tensors, it is now possible to draw a picture of the local ordering at several length scales across the film. In the interfacial region, the backbone bonds of the chains orient parallel to the solid surface while the side groups are perpendicularly ordered. The parallel orientation in the backbone is persistent up to three C–C bonds. The chains were shown to be compressed in the *z* direction due to the presence of the solid surfaces and were aligned in the *xy*-plane parallel to the surface overall. Moving into the bulk of the film, the local bond ordering vanishes and all C–C bonds depict random orientation patterns. On a larger length scale, the chains show unperturbed structures in the *z* direction while still slightly flattened in the *xy*-plane parallel to the surface. As a result, a systematic structural profile is developed throughout the film due to the confinement which is absent in the bulk simulations. These results are in agreement with previous studies on other interfacial systems including PE/graphite [12], PP/graphite [54], acrylic polymers/α-quartz, as well as acrylic polymers/α-Fe_2_O_3_ [26].

### 3.3. Surface Tension Evaluation

In this part of the manuscript, we set out to provide data on the surface tension, γ, of our confined model. The hydrodynamic definition of surface tension is the isothermal work of formation per unit area of interface. At the atomic scale, it can be expressed as the integrated imbalance of normal and tangential pressures, $P_{N}(z)$ and $P_{T}(z)$ respectively, at the interface as [15,16]

(6) $$\gamma =\frac{1}{2}\int_{-\infty }^{+\infty }\left[P_{N}{(z)-P}_{T}(z)\right]dz.$$

**Figure 9:**
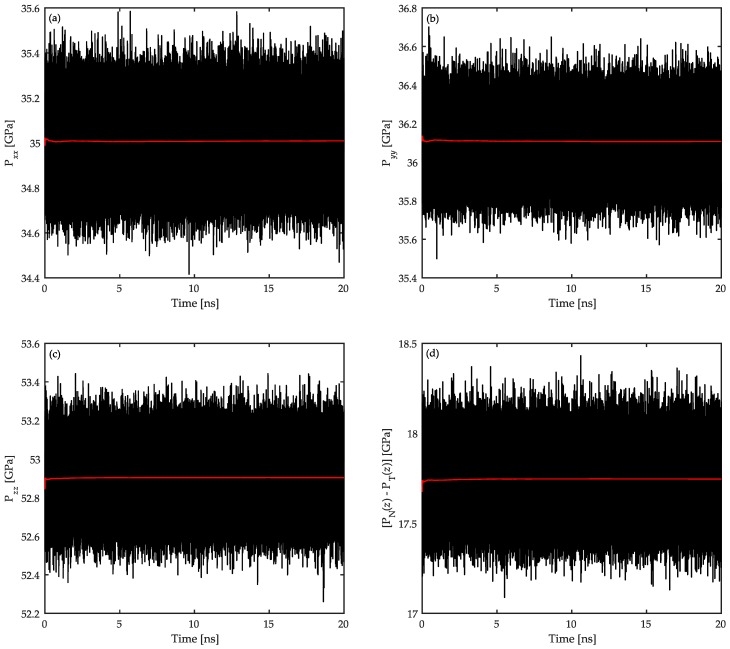
Instantaneous (**black** curve) and running average (**red** curve) profiles of (**a**) $P_{\mathrm{xx}}(z)$; (**b**) $P_{\mathrm{yy}}(z)$; (**c**) $P_{\mathrm{zz}}(z)$; and (**d**) $\left[P_{N}{(z)-P}_{T}(z)\right]$. These data are calculated for polymer atoms in the interfacial regions of the confined PP/Fe_2_O_3_ system at 458 K and 1 atm.

For a plane interface in our study which is perpendicular to the *z* direction, the tangential and normal pressures are defined as

(7) $$P_{N}{(z)=P}_{\mathrm{zz}}(z),$$

and

(8) $$P_{T}(z)=\frac{1}{2}\left[P_{\mathrm{xx}}{(z)+P}_{\mathrm{yy}}(z)\right],$$

respectively. In these equations, the components of the pressure tensor are calculated using the virial theorem of statistical mechanics according to

(9) $$P_{\alpha \alpha }=\langle \frac{1}{V}\left[\underset{j}{\sum }m_{j}v_{\alpha j}^{2}+\underset{i>j}{\sum }\underset{j}{\sum }r_{\alpha \mathrm{ij}}f_{\alpha \mathrm{ij}}\right]\rangle ,$$

with $m_{j}$, $v_{j}$, $r_{j}$, and $f_{j}$ representing the mass, the velocity vector, the position vector, and the total force acting on the jth atom, respectively. Here, V denotes the volume of the thin layers parallel to the *xy*-plane each with a thickness of dz. Typical plots of the time evolution of the three diagonal components of the pressure tensor as well as the corresponding $\left[P_{N}{(z)-P}_{T}(z)\right]$ values are shown in Figure 9. The data are calculated for polymer atoms in the interfacial regions of the confined PP/Fe_2_O_3_ system at 458 K and 1 atm. In the figures, black and red curves show the instantaneous and running average values, respectively. Since the normal pressure has reached a constant value in the interfacial region (see Figure 9c and note Equation (7)), and $P_{N}{(z)=P}_{T}(z)$ further away from the interfaces, one can conclude that the system is at equilibrium [15]. As evidenced by the pressure profiles, the oscillations in the pressure during the data collection simulations are negligible in the NVT ensemble. This consistency in the pressure profile is mainly due to the effectiveness of the relaxation simulations at high temperatures performed before running the data collection simulations. Such a negligible oscillation is better highlighted if one considers the more pronounced pressure oscillations in the similar study of Anastassiou et al. [26] who utilized the NPT ensemble in their simulations. This comparison as well as previous arguments ensure that the studied system is well-equilibrated and the NVT ensemble in this work produces precise thermodynamic data with minor influence from the pressure oscillations. The use of the NVT ensemble was also motivated in this study in order to control the degree of confinement by preserving a constant volume throughout the simulations.

**Figure 10:**
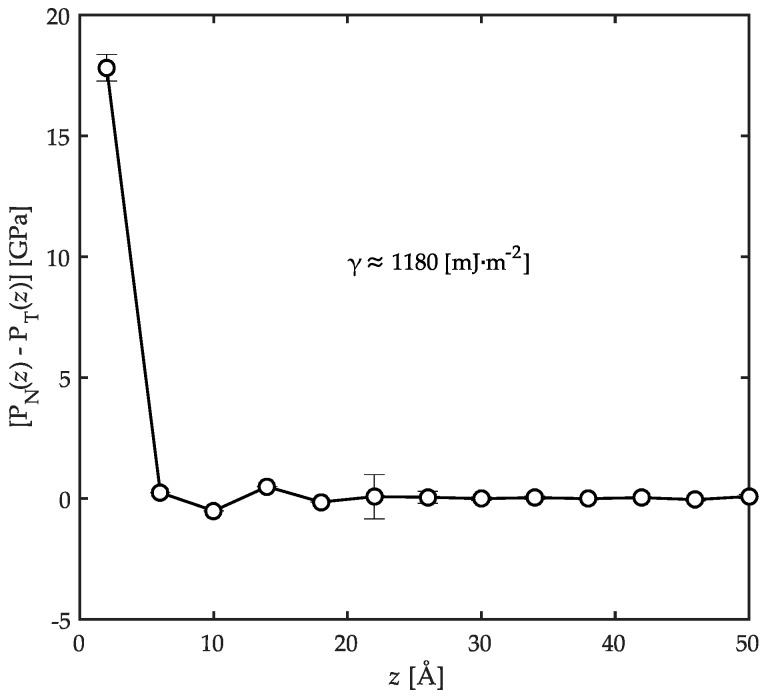
The profile of the difference of normal $\left(P_{N}(z)\right)$ and tangential pressures $\left(P_{T}(z)\right)$ across the film thickness for the confined PP/Fe_2_O_3_ system at 458 K and 1 atm. The surface tension is calculated from Equation (6) to be approximately equal to ~1180 mJ·m^−2^.

In order to utilize Equation (6), we need to evaluate the profile of $\left[P_{N}{(z)-P}_{T}(z)\right]$ across the thickness of the film. To do this, the film was divided into bins each with a 4 Å thickness and the pressures were monitored for all the atoms in the corresponding bins. The results are shown in Figure 10. According to the pressure profiles, the intrinsically anisotropic nature of the local pressure tensor extends over distances up to approximately 18 Å away from the solid surface. Beyond this distance, the pressure tensor becomes isotropic indicating that the presence of the substrate is not felt there. This is another indication that the structural and conformational properties of the bulk of the film depict the corresponding properties of the unconfined bulk polymers.

Benefitting from Figure 10, the surface tension is calculated utilizing Equation (6) to be approximately equal to ~1180 mJ·m^−2^. The hydrodynamic definition of surface tension is the isothermal work of formation/destruction per unit area of interface. This value was shown before to be the main constituent part of the work of adhesion (or the adsorption energy) in interfacial systems [21,26,43,44,48,54]. It signifies a stronger interfacial adhesion between the components as it becomes larger, after the interface is formed under confinement due to space-filling considerations [26]. Considering typical surface energy values for Fe_2_O_3_ in the range of 2250 to 2360 mJ·m^−2^ [26,34], and for PP approximately 20.29 mJ·m^−2^ [58,59], it can be concluded that the formed interface with the evaluated energy per unit area under this confined conditions is strongly adherent. This adsorption is effective up to a depth of 18 Å into the film thickness from the solid surface. For PP/Fe_2_O_3_ interfaces at the simulated temperature of 458 K, we were not able to find any experimental reports in the literature on the surface tension value probably due to difficulties in the experimentation procedure. Consequently, direct comparison of this result with experiments is not possible at this time. However, it cannot be totally wrong to expect that the theoretical surface tension should be significantly different from probable measurements. In a similar study on polymer/solid interfaces, Anastassiou et al. [26] also reported such a significant difference and ascribed it to several factors such as (i) the very smooth atomic nature of the substrates in simulations; (ii) the perfect crystalline structure assumed for the simulated substrates; and (iii) the absence of any impurities at the interfaces in simulations. In experiments on the other hand, surface roughness, imperfect crystalline structures of Fe_2_O_3_ substrates, and contamination influence the results to a large extent. The authors also report the values for the adsorption energy of acrylic adhesives on α-Fe_2_O_3_ based on the same approach used here to be ~1654 mJ·m^−2^. The authors discussed in detail that as the adsorption energy increases, the adhesion strength is improved. This further supports the earlier conclusion that the interfaces between PP and Fe_2_O_3_ with an interfacial energy of ~1180 mJ·m^−2^ are strongly adherent after formation under confinement. Moreover, from a practical viewpoint, the SDS process could not be properly carried out if this notion was not true. Our experience shows that it is possible to perform SDS with PP/Fe_2_O_3_ systems [6].

It should be noted that the methodology of surface tension evaluation used here has been successfully applied to other interfacial systems for which experimental results are also available. Mansfield et al. [48] used this approach and evaluated the surface energy of a glassy atactic PP exposed to vacuum at 233 K. Their theoretical prediction of the surface energy of 43 ± 23 mJ·m^−2^ was in good agreement with the experimental value of 45.9 ± 2 mJ·m^−2^. The authors further applied this technique to calculate the surface tension of this PP under the same conditions with a graphite substrate [54]. In this way, they could successfully re-produce the experimental value for the work of adhesion. Alejandre et al. [15] applied this approach to study the liquid-vapor equilibrium of water and successfully calculated the surface tension of water for a range of temperatures from 316 to 573 K. A similar study on water was also carried out by Shi et al. [16]. These works are valuable proofs that the current methodology for surface tension evaluation is effective.

## 4. Conclusions

In this study, atomistic MD simulations of confined molten PP chains between Fe_2_O_3_ surfaces were performed. Local structures formed in the polymer film were studied utilizing density profiles, orientation of bonds, and end-to-end distance of chains. The density profile proved the existence of an adsorbed polymer layer on the surface with a thickness of ~7 Å. At distances beyond 20 Å into the film, the density was similar to that of unconfined PP. Based on previous works, it was argued that this distance is mainly determined by thermal motions rather than chain size. The number of adsorbed carbon and hydrogen atoms was also estimated at interfacial regions. It was observed that most of the adsorbed atoms (~70%) are closely packed on the oxide surface rather than freely dangling in the bulk of the film. This was ascribed to the strong adsorption of polymer chains on the surface as a result of the high surface energy of the metal oxide substrates.

The local order parameter and the conformation tensors were calculated in order to have an idea of the locally-ordered structures of confined PP chains at various length scales. This analysis revealed that at the interfacial region, the backbone C–C bonds of the chains orient parallel to the solid surface while the C–C bonds with the side groups tend to perpendicularly orient to a limited degree. The order parameter in the interfacial regions was discussed to be dominated by space-filling considerations. For both C–C bond types, random orientation was observed in the bulk of the film. In general, the PP chains prefer to flatten in the *xy*-planes parallel to the solid surfaces on a larger length scale. This behavior is more pronounced at the interfacial regions compared to the bulk of the film.

Finally, the surface tension was calculated utilizing the buildup imbalance of normal and tangential pressures. The surface tension value reveals a strong adsorption of PP chains on the Fe_2_O_3_ substrate after they formed an interface under confinement. It was observed that the intrinsically anisotropic nature of the local pressure tensor extends over distances up to approximately 18 Å away from the solid surface. Beyond this distance, the pressure tensor becomes isotropic indicating that the presence of the substrate is not felt there.

These results confirm the applicability of PP as a backbone component in a MIM feedstock containing ferritic materials due to the strong adsorption and parallel orientation of the polymer at the surface of Fe_2_O_3_ substrates. This notion is particularly important since the backbone polymer should hold the metal particles in place during the shaping process, and while other components are being removed during the debinding step. This guarantees that the geometry of the molded part will be maintained and sintering can be done to obtain a metal part with complex shape.
